# Risk factors for recurrent wheezing after infant bronchiolitis: a 6-year single-centre follow up study in China

**DOI:** 10.3389/fped.2025.1549475

**Published:** 2025-08-07

**Authors:** Sainan Chen, Xueyun Xu, Min Wu, Luting Zhou, Yuqing Wang

**Affiliations:** Department of Respiratory Medicine, Children’s Hospital of Soochow University, Suzhou, China

**Keywords:** bronchiolitis, recurrent wheezing, risk factors, eczema, RSV

## Abstract

**Background:**

Infants with bronchiolitis have an increased risk of developing recurrent wheezing and asthma. However, the risk factors for recurrent wheezing or asthma after infant bronchiolitis remain controversial. The aim of our prospective observational study was to seek the risk factors for recurrent wheezing or asthma.

**Methods:**

Infants with first bronchiolitis attack at the respiratory department, Children's Hospital of Soochow University were enrolled from November 2016 through March 2017. Serum cytokines, TSLP, IL2, IL13, TIMP-1, MMP-9, IL33, IL5, IL4, IL25, TNF-α and MIP-1α were measured via flow cytometry at enrolment. Patients were followed up with every 3 months for a duration of 6 years by telephone or as outpatients for the number of wheezing episodes. In the sixth year of follow-up, lung function tests, total IgE and allergen specific IgE test were performed in those children at 6–7 years of age.

**Results:**

We enrolled 89 infants, 72 of whom were successfully followed up for 6 years. In total, 31.9% of the patients developed recurrent wheezing and 12.5% of patients developed asthma after 6 years of follow-up. The Kaplan–Meier curves of the overall analytic cohort (*n* = 72) revealed that compared with those in the non-eczema group and non-RSV group, the rate of recurrent wheezing preschool was significantly higher in patients with bronchiolitis with eczema and RSV (*P* < 0.05). There were no significant differences in cytokine levels between patients with and without current asthma (*P* > 0.05).

**Conclusions:**

A total of 31.9% of the children with hospitalization for bronchiolitis at an early age developed recurrent wheezing and 12.5% developed asthma at 6-years old. Infants hospitalised with RSV bronchiolitis and/ or with a history of eczema were at increased risk for developing recurrent wheezing.

## Introduction

Acute bronchiolitis is a common lower respiratory tract disease characterized by wheezing, cough, tachypnea and chest retractions (1). It is often caused by viral infection in infants under two years of age and respiratory syncytial virus (RSV) is the most common cause (2).

The relationships between bronchiolitis and recurrent wheezing/asthma have been reported in many studies (3–5). The bronchioles are completely or partially blocked by cell debris, small airway resistance increases significantly and the lung compliance decreases. Most children have a good prognosis, but recurrent wheezing or even asthma may occur in later life due to airway hyperresponsiveness and progressive pulmonary dysfunction after RSV infection of the lower respiratory tract (3). Multicentre follow-up data from the USA revealed that the incidence of postbronchiolitis recurrent wheezing was as high as 31% (4). In a follow-up study in China, approximately 35.1% (26/74) of infants with RSV-associated bronchiolitis aged 6 months or younger experienced recurrent wheezing at the age of 3 years (5).

In previous studies, the risk of postbronchiolitis recurrent wheezing/asthma has been linked to rhinoviruses and RSV (5–7). In addition, prematurity, family history of asthma, passive smoking, atopic dermatitis and disease severity in infancy have been identified as risk factors for post bronchiolitis recurrent wheezing or asthma (8–11). In recent years, increasing attention has been given to the relationship between cytokine levels and postbronchiolitis asthma (7, 12).

There are many follow-up studies on the relationship between asthma or recurrent wheezing and bronchiolitis. In contrast, research in developing countries such as China are limited. In addition, asthma is a heterogeneous disease, which is affected by genetics, the environment and economic development level, that leads to regional differences in the incidence of asthma. Few long-term follow-up studies have analysed the impact of bronchiolitis on recurrent wheezing/asthma in China. We prospectively followed children who were hospitalised for bronchiolitis at less than two years of age for 6 years. The aim of the present study was to evaluate the long-term outcomes of children who were hospitalised for bronchiolitis in the early stage of life at 6–7 years of age. In addition, we studied the impact of early-life risk factors, such as atopic dermatitis, family history of asthma, exposure to tobacco smoke, the viral aetiology of bronchiolitis, and serum cytokines, on patient asthma or recurrent wheezing.

## Methods

### Study population

Infants who were diagnosed with bronchiolitis and hospitalised at the Department of Respiratory Disease at Children's Hospital Soochow University, China, from November 2016 through March 2017, were enrolled in this cohort study. Informed consent was obatined from all guardians of partcipants. The study was approved by the Medical Ethics Committee of Children's Hospital Affiliated to Soochow University (Approval No.: 2016050).

The inclusion criteria were as follows: (1) patients aged 1–24 months; (2) patients were hospitalised with bronchiolitis; (3) bronchiolitis was defined as the first wheezing episode characterized by cough, tachypnea and chest retractions.

The exclusion criteria were as follows: neuromuscular disease, congenital airway deformity, congenital heart disease, gastroesophageal reflux disease, foreign body inhalation, primary or secondary immune deficiency or other immune-associated diseases.

Disease severity criteria: according to Wang expiratory flow limitation (EFL) scoring (13), the severity of disease was graded as follows (Table 1): 0–4.9 was mild; 5–8.9 was moderate; 9–12 was severe.

**Table 1:** Scoring standard of bronchiolitis.

Symptoms	0	1	2	3
R(times/min)	<30	31–45	46–60	>60
wheezing	No	Only can be heard at the end of expiration by stethoscope	Be heard during expiration with or without stethoscope	Be heard during inspiration and expiration without stethoscope
Tri-retraction sign	No	Only rib gap depression	Trachea depression	Nasal flaring
Mental condition	Normal			Irritability, drowsiness and decreased feeding

### Sample collection and detection

Nasopharyngeal aspirates were obtained from all inpatients within 24 h of admission. Seven common viruses, including respiratory syncytial virus (RSV), influenza virus (IVA, IVB), parainfluenza virus (PIV1∼3) and adenovirus (ADV) were detected via direct immunofluorescence (DFA) (14). The nucleic acid extracts were tested for human rhinovirus (HRV), human metapneumovirus (HMPV) and bocavirus (HBoV) via fluorescent quantitative PCR and RT‒PCR (14). During their admission, peripheral venous blood (2 ml) was also collected for detection of routine blood parameters, humoral and cellular immunity and cytokines (15). Serum cytokines including thymic stromal lymphopoietin (TSLP), interleukin 2 (IL2), interleukin (IL13), tissue inhibitor of metal protease 1 (TIMP-1), matrix metalloproteinase (MMP-9), interleukin 33 (IL33), interleukin 5 (IL5), interleukin 4 (IL4), interleukin25 (IL25), tumor necrosis factor- α (TNF- α) and macrophage inflammatory protein-1α (MIP-1α) were measured via flow cytometry (15).

### Data collection

Each patient's data, including age, sex, gestational age at delivery, birth weight, feeding pattern, history of eczema, family history of asthma, exposure tobacco smoke, pet contact and all the blood results at admission were recorded.

### Follow-up of patients

After discharge from the hospital, the patients were followed up every 3 months for a 6-year period via outpatient visits or telephone consultations. At the sixth year of follow-up, pulmonary function tests, total IgE and allergen specific IgE test were performed. Lung function was measured by spirometry, using Master Screen spirometer (Germany). Variables recorded were forced vital capacity (FVC), forced expiratory volume in 1 s (FEV1), FEV1/FVC- ratio and forced expiratory flow between 25% and 75% of the FVC (MEF25-75), and presented as percentage of predicted. Bronchial dilation test is a review of lung function after half an hour of inhalation of β-receptor agonists in the presence of airway obstruction. Positive bronchodilation test refers to a 12% increase in FEV1. RAST test was used to detect allergens. Allergens include inhaled allergens (dust mites, Aspergillus fumigatus, cat dander, dog dander, cockroaches, pollen) and ingested allergens (egg white, milk, wheat, peanuts, soybeans, crabs, shrimp, nuts).

The definition of recurrent wheezing was wheezing ≥ 3 times, with each interval between at least one week or more.

The definition of asthma was as follows (16, 17): current asthma was considered present if the child had experienced repeated wheezing and inhalation therapy was effective, or reversible airflow limitation was also found in the pulmonary function test.

### Statistical analysis

SPSS version 18.0 software was used for data analysis. The normality of the distribution of continuous data was tested via P-P plots methods before comparison. Data with a normal distribution were presented as the mean ± standard deviation (SD) and were analysed via t tests. Continuous data with a non-normal distribution were represented as the median (minimum-maximum) and were analysed with the Mann–Whitney *U*-test. Categorical data were represented as frequency and were analysed with Chi square examination. Survival analysis was used to compare the difference of recurrent wheezing between eczema group and non-eczema group.

## Results

### Follow-up results

In all, 89 children (56 males and 33 females; mean age: 4.68 ± 4.37 months) hospitalised for bronchiolitis under the age of two years old participated in the follow-up study. Of those, 72 children (47 males and 25 females; mean age at enrollment: 4.65 ± 3.93 months) were successfully followed up for six years which were achieved through telephone interview or clinic visit.

**Figure 1:**
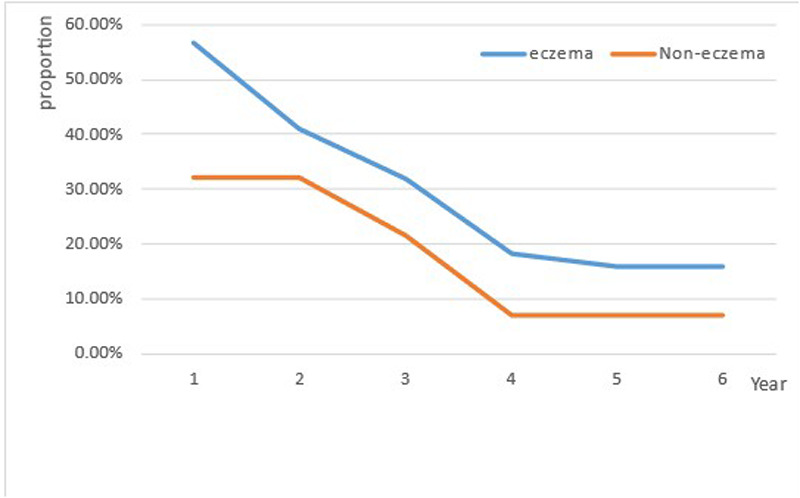
The proportion trend in the incidence of wheezing over with increasing age in children with or without eczema.

**Figure 2:**
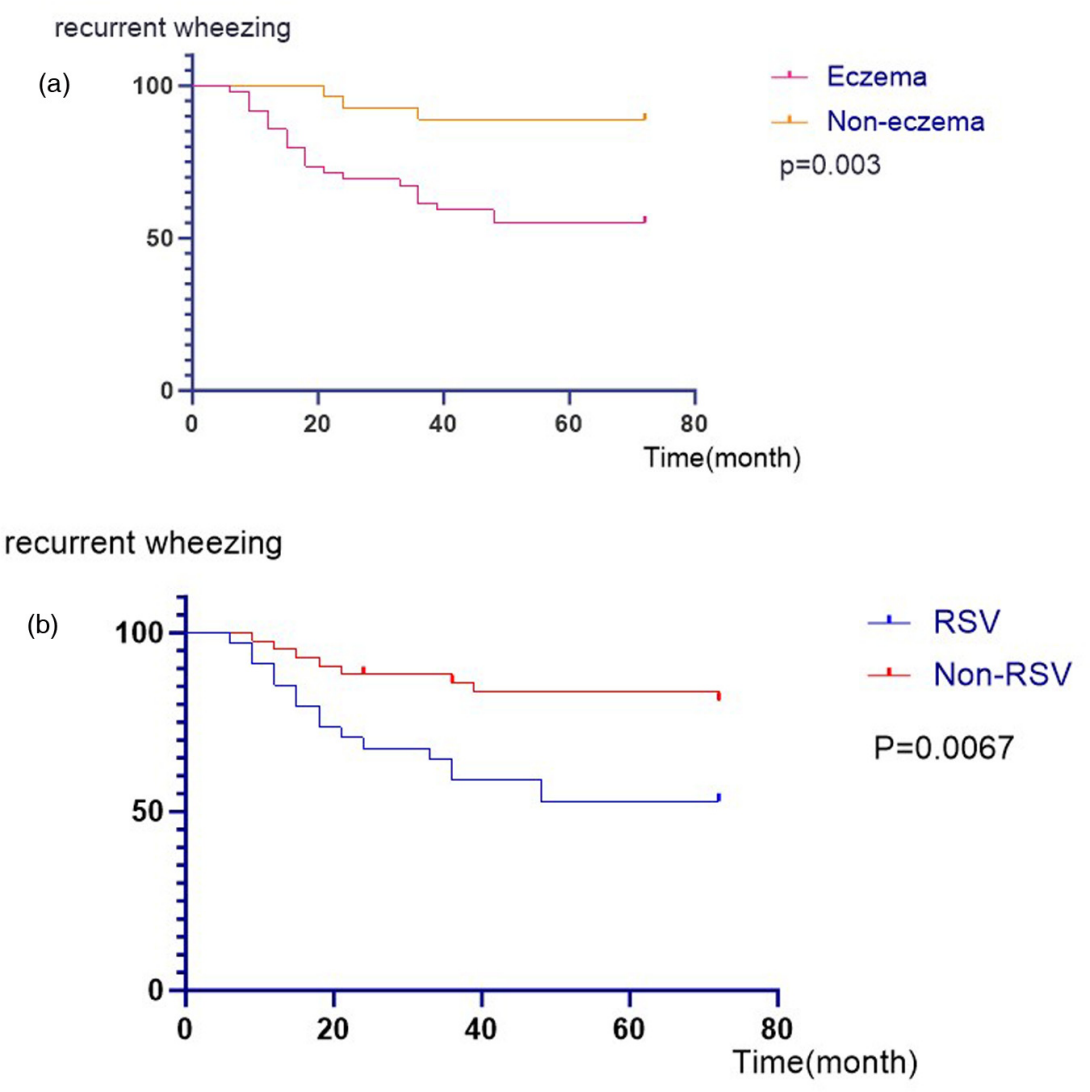
**(a)** Kaplan–Meier curves for recurrent wheezing after bronchiolitis hospitalization between eczema group and the non-eczema group. **(b)** Kaplan–Meier curves for recurrent wheezing after bronchiolitis hospitalization between the RSV group and the non-RSV group.

In the sixth year of follow-up, 30 children completed the pulmonary function tests, allergen detection and total IgE assessment. Among them, 70% (21/30) patients had normal lung function, 6.7% (2/30) patients had small airway dysfunction, 13.3% (4/30) patients had decreased MEF25, 10% (3/30) patients had mild obstructive pulmonary ventilation disorder, of which 6.7% (2/30) patients had negative bronchial dilation tests, and 3.3% (1/30) patient had positive bronchial dilation test. Inhaled allergens were detected in 8 children. In total, 31.9% of the patients developed recurrent wheezing. Current asthma was present in 9 (12.5%) children, 7 (77.8%) of whom had eczema (Table 2). The percentage of wheezing in children with eczema was 15. 9%, and in children without eczema, it was 7.1% (Figure 1). The Kaplan–Meier curves of the overall analytic cohort (*n* = 72) revealed that compared with those of the non-eczema group and non-RSV group, the rate of recurrent wheezing preschool was significantly greater in patients with bronchiolitis with eczema and/or RSV (*P* < 0.05) (Figures 2a,b).

**Table 2:** Virus distribution in the 72 patients with bronchiolitis.

Virus	Total, *n* = 72 no. (%)	Asthma group, *n* = 9 no. (%)	Non-asthma group, *n* = 63 no. (%)
RSV	34 (47.2)	7 (77.8)	27 (42.8)
HRV	3 (4.2)	1 (11.1)	2 (3.2)
Metapneumovirus	0 (0)	0 (0)	0 (0)
HBoV	2 (2.8)	0 (0)	2 (3.2)
Parainfluenza virus	0 (0)	0 (0)	0 (0)
Influenza virus	0 (0)	0 (0)	0 (0)
Adenovirus	0 (0)	0 (0)	0 (0)
Not detected	35 (48.6)	2 (28.6)	33 (52.4)
RSV + HRV	2 (2.8)	1	1

### Virus detection rate

In the 72 patients with bronchiolitis, the detection rate for respiratory viruses was 50.6%. The most common pathogen was RSV (47.2%), followed by HRV (4.2%) and HBoV (2.8%). Two patients (2.5%) had coinfections with RSV and HRV (Table 2).

### General characteristics

There were no significant differences between the asthma group and the non-asthma group with respect to sex, age, premature birth, low birth weight, feeding patterns, eczema, family history of asthma or others (*P* > 0.05) (Table 3).

**Table 3:** Comparison of baseline data between children with asthma and without asthma.

Basic characteristics	Asthma group *N* = 9		Non-asthma group *N* = 63		*P* value
	*n*	%	*n*	%	
Sex(male)	3	33.3	44	63.8	0.075^a^
Age (≤ 6 months)	7	77.8	46	73	1.000^a^
Premature birth	0	0	1	1.6	1.000^b^
Low birth weight	1	11.1	2	3.2	0.824^a^
Macrosomia	1	11.1	8	12.7	1.000^a^
Feeding patterns					
Breast feeding	7	77.8	36	57.1	0.414^a^
Artificial feeding	0	0	8	12.7	0.584^b^
Mixed feeding	2	22.2	19	30.2	0.922^a^
Eczema	7	77.8	37	58.7	0.465^a^
Family history of asthma	2	22.2	4	6.3	0.334^a^
Exposure to smoking	4	44.4	30	47.6	1.000^a^
Pet contact	1	11.1	1	1.6	0.588^a^

^a^ Continuous correction.

^b^ Fisher exact probability method.

### Clinical characteristics

There were no significant differences between the asthma group and the non-asthma group with respect to fever, feeding difficulty, chest retractions or severe conditions (*P* > 0.05) (Table 4).

**Table 4:** Comparison of clinical characteristics between children with asthma and without asthma.

Clinical characteristics	Asthma group *N* = 9		Non-asthma group *N* = 63		*P* value
	*n*	%	*n*	%	
Fever	0	0	6	9.5	0.435^b^
Feeding difficulty	2	22.2	14	22.2	0.747^a^
R > 60 times/min	0	0	5	7.9	0.502^b^
Chest retractions	2	22.2	10	15.9	1.000^a^
Cyanosis	0	0	4	6.3	0.579^a^
Severe condition	2	22.2	12	19.0	1.000^a^

^a^ Continuous correction.

^b^ Fisher exact probability method.

### Laboratory examinations

There were no significant differences between the asthma group and the non-asthma group in terms of blood cell count, humoral immunity, cellular immunity, serum total IgE, antigen-specific IgE or RSV infection (*P* > 0.05) (Table 5).

**Table 5:** Comparison of laboratory examinations between children with asthma and without asthma.

Laboratory examinations	Asthma group *N* = 9		Non-asthma group *N* = 63		*P* value
Blood cell count (×10^9^/L)					
White blood cell	8.34 ± 2.58		10.41 ± 3.99		0.137^b^
Absolute lymphocyte count	5.21 ± 2.69		6.41 ± 2.92		0.248^b^
Absolute neutrophil count	2.40 ± 1.46		2.97 ± 2.18		0.451^b^
Eosinophil count	0.06 ± 0.11		0.11 ± 0.15		0.361^b^
Platelet count	449.3 ± 121.9		445.1 ± 130.1		0.927^b^
Humoral immunity (g/L)					
IgA	0.15 (0.01,0.35)		0.19 (0.01,1.03)		0.438^a^
IgG	3.92 (2.44,5.04)		4.91 (3.76,10.59)		0.102^a^
IgM	0.52 ± 0.24		0.67 ± 0.35		0.252^b^
Cellular immunity					
CD3+	0.63 ± 0.11		0.63 ± 0.10		0.959^b^
CD4+	0.41 ± 0.11		0.42 ± 0.10		0.855^b^
CD8+	0.18 ± 0.09		0.19 ± 0.06		0.787^b^
NK	0.12 ± 0.07		0.13 ± 0.07		0.693^b^
CD19+/CD23+	0.13 ± 0.06		0.12 ± 0.06		0.876^b^
Total IgE (IU/ml)	15.5 (2.3,66.6)		25.5 (0.2,367)		0.834^a^
Antigen-specific IgE					
Milk protein	1	11.1	5	7.9	1.000^c^
Egg protein	1	11.1	3	4.8	1.000^c^
RSV (n, %)	7	77.8	27	42.8	0.108^d^

^a^ *t*-test.

^b^ Nonparametric rank sum test.

^c^ Fisher exact probability method.

^d^ Continuous correction.

^e^ RAST positive means that the content of specific IgE in serum is more than 3.5 times higher than the average value of normal people.

### Serum levels of cytokines

**Figure 3:**
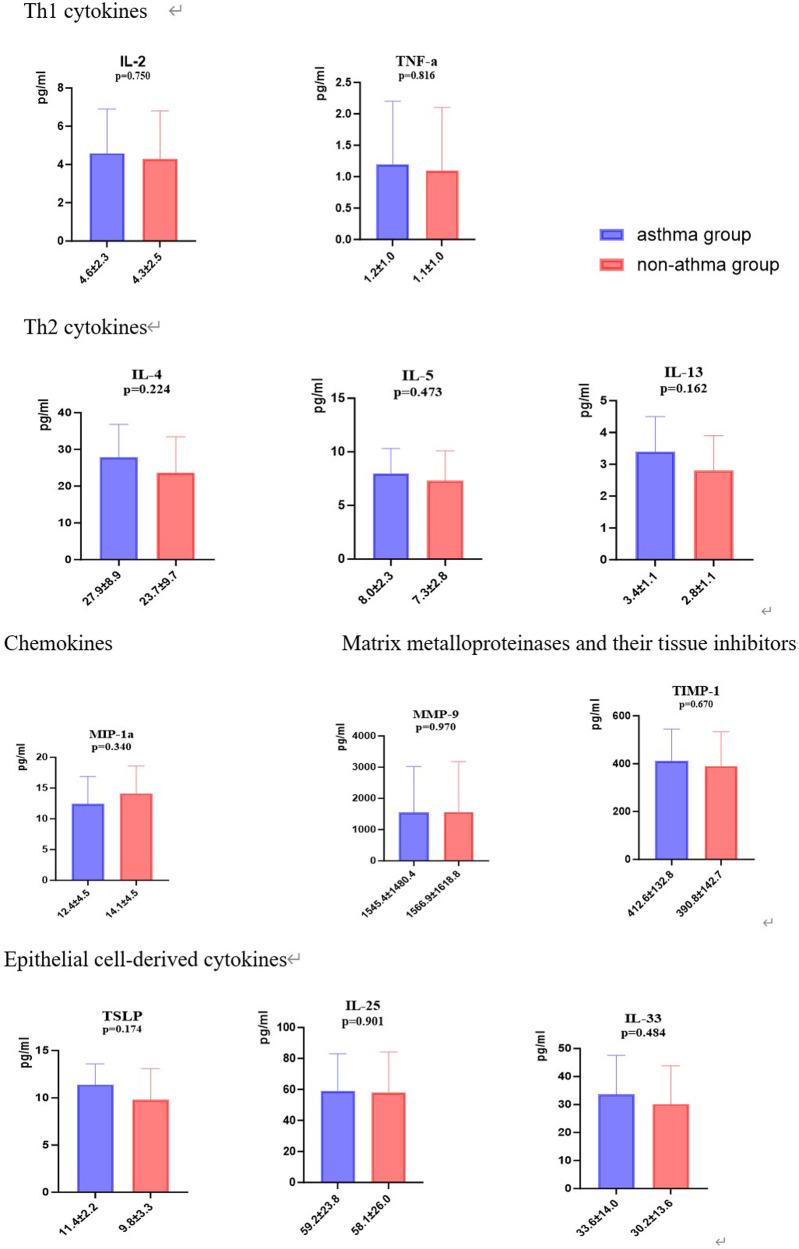
Comparison of serum level of cytokines between patients with and without asthma.

There were no significant differences between patients in the asthma group and the non-asthma group regarding the

levels of TSLP, IL2, IL13, TIMP-1, MMP-9, IL33, IL5, IL4, IL25, TNF-α and MIP-1α (*P* > 0.05) (Figure 3).

## Discussion

In this study, 72 children were successfully followed up for six years which was achieved by a telephone interview or clinical visit. Among those, 31.9% of the children developed recurrent wheezing and 12.5% were diagnosed with asthma in the sixth year of follow-up. Infants hospitalised with RSV bronchiolitis and/or with a history of eczema were at increased risk for developing recurrent wheezing.

Recent studies have shown that 15%–24% of infants hospitalised for bronchiolitis subsequently develop asthma in childhood (18–22). A 7-year follow-up study in Zhejiang, China revealed that the prevalence of post-RSV-bronchiolitis asthma among 266 infants was approximately 24% (21). Makiko Nanishi et al. followed up with 1,130 infants hospitalised for bronchiolitis and reported that 15% subsequently developed asthma by the age of 12 years (19). Our results show that the incidence of asthma is slightly lower. The apparent discrepancies between these reports may be attributable to the differences in the study design, regional races, sample, outcome definitions, or any combination of these factors. Few long-term follow-up studies have analysed the impact of bronchiolitis on recurrent wheezing/asthma in China. Our follow-up study lasted for 6 years, during which we followed up every 3 months. The recall bias was smaller than that of other domestic studies, and the data were relatively reliable.

While the incidence of wheezing declines overall with age, in addition to transient wheezing, children with eczema present persistent wheezing or later asthma. In our previous study (15), a history of eczema was the only independent risk factor identified in children with postbronchiolitis recurrent wheezing. In this study, infants with a history of eczema had a greater risk of developing recurrent wheezing but not asthma. Several studies have shown that recurrent wheezing in infants is related to eczema (23, 24). A multicentre study in the United States revealed that the risk of recurrent wheezing was significantly increased in children with eczema (25). A retrospective study of 167 children with bronchiolitis in Chengdu (26), China showed that children with allergies had a significantly increased risk of recurrent wheezing. Eosinophils play a key role in the pathogenesis and development of atopic diseases including asthma and eczema (27). Our study found that eczema was associated with recurrent wheezing after bronchiolitis, but not with asthma. That may be due to our small sample size.

Some researchers have attributed the mechanisms underlying the associations between bronchiolitis and the development of asthma to virus infection (6, 7, 28, 29). It is still controversial whether the virus causes recurrent wheezing or asthma after bronchiolitis and which virus infection causes postbronchiolitis recurrent wheezing/asthma. A number of previous studies have reported an association between RSV infection in early childhood and an increased incidence of subsequent asthma (6, 7, 21, 30–33). For example, a population-level study of more than 740,000 children reported an increased risk of asthma after RSV-hospitalization (6). In contrast, other studies have shown that children hospitalised with bronchiolitis caused by other viruses develop recurrent wheezing/asthma at higher rates than children with RSV-bronchiolitis (20, 29, 34). A 17- centre multicenter severe bronchiolitis cohort study revealed that infants who were hospitalised with HRV infection accompanied by new HRV infection had the highest risk of recurrent wheezing (35). In our study, we found no significant correlation between virus infection and asthma after bronchiolitis, although the RSV infection rate in the asthma group was 77. 8% (7/9). The reason for this result may be the insufficient sample size and different detection rates of viruses in children. The RSV/HRV rate in our study was lower than that in the above studies, which may be related to the test method (DFA) being associated with variable and lower sensitivity than PCR. However, we found that children with RSV bronchiolitis have a greater risk of recurrent wheezing. This may be due to the patients with RSV bronchiolitis developed more severe illness in comparison with patients with bronchiolitis due to other respiratory viruses (36). In this situation, further study is needed to ascertain the correlation between viral infection and asthma after bronchiolitis.

Our study investigated the influence of various serum inflammatory cytokines including Th1 cytokines (IL2, TNF-α), Th2 cytokines (IL4, IL5, and IL13), chemokine (MIP-1α), matrix metalloproteinases and their tissue inhibitors (TIMP-1 and MMP-9) and epithelial cell-drived cytokines (TSLP, IL25, and IL33), on the subsequent development of asthma after hospitalization for bronchiolitis in early life. No serum cytokines were found to be associated with asthma after bronchiolitis. In contrast, in a 17-centre prospective cohort study of infants with severe bronchiolitis, serum periostin induced by type 2 inflammatory cytokines (e.g., interleukin IL-4 and IL-13) was associated with asthma risk by age 6 years (37). Another multicentre prospective study reported that higher nasopharyngeal airway type 2 cytokine (IL-4, IL-5, IL-13, and TSLP) levels in infants with HRV infection alone were associated with a greater risk of developing childhood asthma (7). A possible explanation is the selection of different specimens and the different definitions and outcomes of asthma. Another reason may be that the sample size of our study is small and it was a single-centre study.

Our study has several limitations. First, we did not have information from healthy controls because our objective was to trace the role of bronchiolitis in early infancy in the development of asthma. Additionally, the sample size was small, the asthma subgroup was small and it was a single-centre study. In addition, our study did not calculate the sample size in advance. Therefore, further multi-centre studies with large sample sizes are needed. On the other hand, we tested cytokines in the serum samples rather than in the nasopharynx aspirates. Changes in cytokine levels in serum play an important role in clinical practice. However, nasopharyngeal secretions may provide more accurate information because they are closer to the site of RSV infection, especially in the early stage of infection, when the viral load may be greater. Finally, our results cannot be extrapolated to mild respiratory infections in an outpatient setting, as all of our patients needed hospital admission. That is, the asthma morbidity observed in our study reflects only the evolution observed in children with bronchiolitis requiring hospitalization.

## Conclusions

A total of 31.9% of the children with hospitalization for bronchiolitis at an early age developed recurrent wheezing and 12.5% developed preschool asthma. Infants hospitalized with RSV bronchiolitis and/ or with a history of eczema are at increased risk for developing recurrent wheezing.

## Data Availability

The data analyzed in this study is subject to the following licenses/restrictions: Due to the privacy issues of the children, the data is not disclosed, and can be applied to the corresponding author if necessary. Requests to access these datasets should be directed to Yuqing Wang, wang_yu_qing@126.com.
